# Waveguide‐Based Four‐Channel Crosstalk‐Free Metahologram Multiplexed by Diffraction Orders

**DOI:** 10.1002/nap2.70078

**Published:** 2026-04-25

**Authors:** Zhiyu Ning, Zeyang Liu, Deqian Zheng, Niu Liu, Hao Gao, Tianzhang Peng, Ziqian Xi, Xinliang Zhang, Cheng Zhang

**Affiliations:** ^1^ School of Optical and Electronic Information Huazhong University of Science and Technology Wuhan China; ^2^ Wuhan National Laboratory for Optoelectronics Huazhong University of Science and Technology Wuhan China

**Keywords:** detour phase, holography, metasurface, waveguide‐based display

## Abstract

Metaholograms have attracted significant attention for their ability to project high‐quality images within a compact form factor. Although various multiplexing strategies have been explored for multi‐channel metaholograms, current approaches still face limitations in display channel extension and inter‐channel crosstalk elimination. Here, we introduce a waveguide‐based metahologram that enables four independent, crosstalk‐free display channels via diffraction‐order multiplexing. The metaholograms are integrated onto the top surface of a flat glass waveguide and modulate guided light through detour phase. With carefully designed device parameters, light diffracted into four distinct orders is selectively out‐coupled to form crosstalk‐free holographic images. To decouple the detour phase modulations between different orders, we develop an adaptive‐weight phase‐retrieval algorithm that simultaneously optimizes phase profiles across multiple diffraction orders to realize independent image encoding. Extending this strategy, we also demonstrate a six‐channel metahologram for two full‐color image projection, highlighting the versatility and scalability of the design. This work offers a new pathway toward compact, high‐capacity holographic devices, with promising applications in AR/VR displays, information storage, and image encryption.

## Graphical abstract

A diffraction‐order‐multiplexed, four‐channel metahologram integrates a flat glass waveguide and an array of titanium dioxide nanopillars to realize crosstalk‐free holographic projection. Light undergoes total internal reflection within the waveguide and gets out‐coupled by diffraction from the nanopillar array. Four independent images (e.g., “Maxwell's four equations” in the figure) can be selectively projected into the far field depending on the diffraction order in the out‐coupling process.

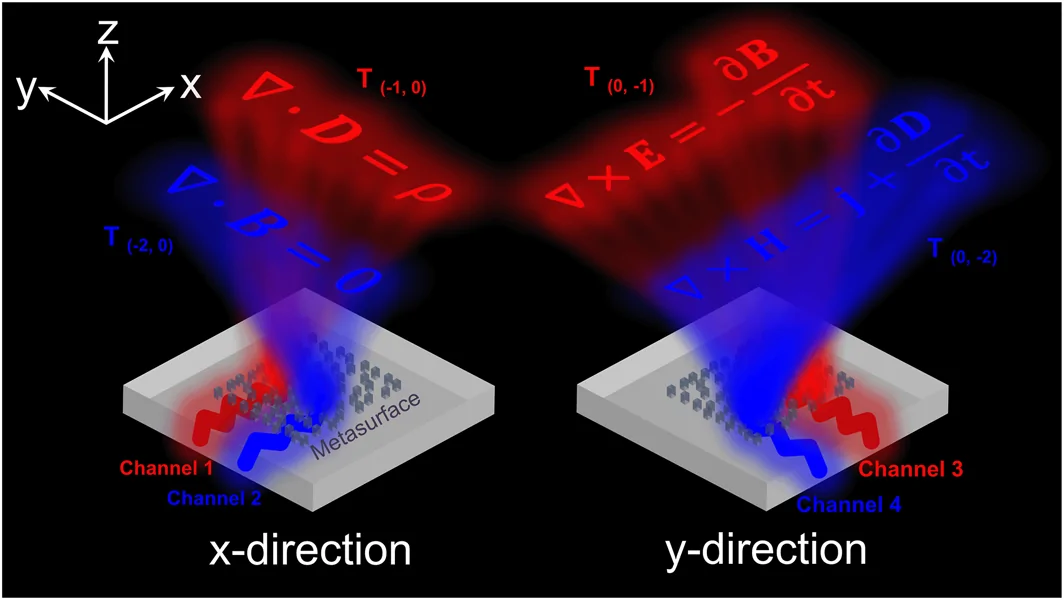

## 1 Introduction

Metasurfaces, as an emerging class of flat optics comparing arrays of artificial subwavelength‐scale meta‐atoms, are capable of flexibly manipulating various state parameters of electromagnetic (EM) waves, including phase, amplitude, and polarization [1, 2, 3, 4, 5, 6, 7]. This versatility enables the creation of numerous compact optical devices for a wide range of applications [8, 9, 10, 11, 12, 13, 14, 15, 16]. Metasurface‐based holograms, also known as metaholograms, can project high‐quality holographic images through proper modulation of an incident light and have shown superiority in spatial resolution, field of view, working efficiency, and operational bandwidth compared to conventional holograms, making them highly attractive for advanced display and imaging applications [17, 18, 19, 20].

Multi‐channel metaholograms, designed using various multiplexing mechanisms, offer the ability to conveniently switch among different projected holographic images by varying the associated multiplexing physical parameter(s) [21, 22]. Therefore, they feature a higher information capacity and are suitable for an array of dynamic display and information storage applications. Over recent years, various multi‐channel metaholograms multiplexed by different state parameters of incident light, such as state of polarization, angle of incidence, wavelength, and orbital angular momentum (OAM), have been proposed [23, 24]. A majority of the proposed designs rely on meta‐atoms that exhibit optical responses sensitive to the variation of a certain state parameter to achieve the multiplexing functionality. Examples include anisotropic meta‐atoms for state of polarization multiplexing [25, 26, 27, 28, 29], U‐shaped meta‐atoms for angle‐of‐incidence multiplexing [30], and high‐aspect‐ratio dielectric meta‐atoms for illumination wavelength multiplexing [31, 32, 33]. Constrained by the underlying physical mechanism, the aforementioned multiplexing methods usually can only provide a limited number of channels (≤ 3 in most cases) for independent holographic projections. A recently proposed approach for multi‐channel metaholograms is based on the *k*‐space translation strategy, which leverages oblique light illumination with different angles of incidence to translate target images in the *k*‐space for selective image projections [34, 35, 36]. Such an approach utilizes the basic property of Fourier transform, thus typically working for holographic projection in the Fraunhofer diffraction region. Also the device suffers from small field of view (FOV) as all target patterns have to be encoded in the evanescent wave region and occupy a small area in the *k*‐space for the purpose of crosstalk elimination. Additionally, noise engineering is recently employed to realize an eleven‐channel polarization‐multiplexed metahologram, but the image quality is compromised by the unavoidable crosstalk among different channels due to the dimension constraints of the Jones matrix [37]. OAM multiplexing enables metaholograms with extended projection channels. However, the projected images inevitably suffer from reduced spatial resolution due to the pixelated spatial sampling for OAM multiplexing and compromised image quality due to the crosstalk among different OAM channels [38, 39, 40]. The cases mentioned above clearly highlight that simultaneously achieving a larger number of independent display channels and effectively suppressing inter‐channel crosstalk remains a challenge for current multi‐channel metahologram designs. It is worth noting that metasurfaces are essentially a type of diffractive optical elements. Apart from the commonly used directly‐transmitted light (zeroth order), multiple higher‐order diffracted light can be generated via proper metasurface design, which offers an additional degree of design freedom and thus provides a potential route toward realizing display channel extension and crosstalk suppression.

Here, we propose a type of diffraction‐order‐multiplexed four‐channel crosstalk‐free metaholograms integrated onto a flat glass waveguide. Incident light propagates inside the glass waveguide by total internal reflection (TIR). Under the illumination of guided incident light with the designed wavelengths and TIR angles, the device is capable of projecting four independent holographic images through four different diffraction‐order channels. To eliminate the inter‐channel crosstalk among different diffraction orders, device parameters are optimized to ensure that only the selected order can be generated in waveguide out‐coupling diffraction under a preset illumination condition. Such metahologram operates based on detour phase modulation, and the holographic phase profiles encoded into different orders are obtained by using an adaptive‐weight phase‐retrieval algorithm. Based on the same design strategy, a diffraction‐order‐multiplexed six‐channel metahologram hosting two sets of RGB display channels has also been demonstrated. This type of waveguide‐based metahologram devices feature enhanced information capacity, compact device size, as well as the capability for multi‐color display and thus show great potential for an array of applications, such as AR/VR display, information storage, and image encryption.

## 2 Device Design and Optimization

The proposed diffraction‐order‐multiplexed four‐channel metahologram consists of a flat glass waveguide and an array of titanium dioxide (TiO_2_) nanopillars with square‐shaped cross‐sections arranged on top. The TiO_2_ nanopillars are identical in size and positioned in square lattices with periodicity of *P* along both the *x*‐ and *y*‐axes. Additionally, each nanopillar exhibits distinct in‐plane displacements along both the *x*‐ and *y*‐axes relative to its corresponding lattice center (see Figure 1). Incident light is conveyed by the flat glass waveguide via TIR. The guided incident light illuminates onto the nanopillar region and subsequently gets out‐coupled from the metahologram by diffraction. By exploiting the displacements of the nanopillars along the *x*‐ and *y*‐axes within their square lattices, detour phase modulations [41, 42] are induced to impart the required holographic phase profiles into four different diffraction orders, which are the minus first and minus second (*m* = −1 and −2) transmission diffraction orders along the *x*‐ and *y*‐axes, denoted as T_(−1, 0)_, T_(−2, 0)_, T_(0, −1)_, and T_(0, −2)_, respectively. In this way, depending on different diffraction orders generated in the out‐coupling process, the metahologram offers four independent holographic projection channels.

**FIGURE 1:**
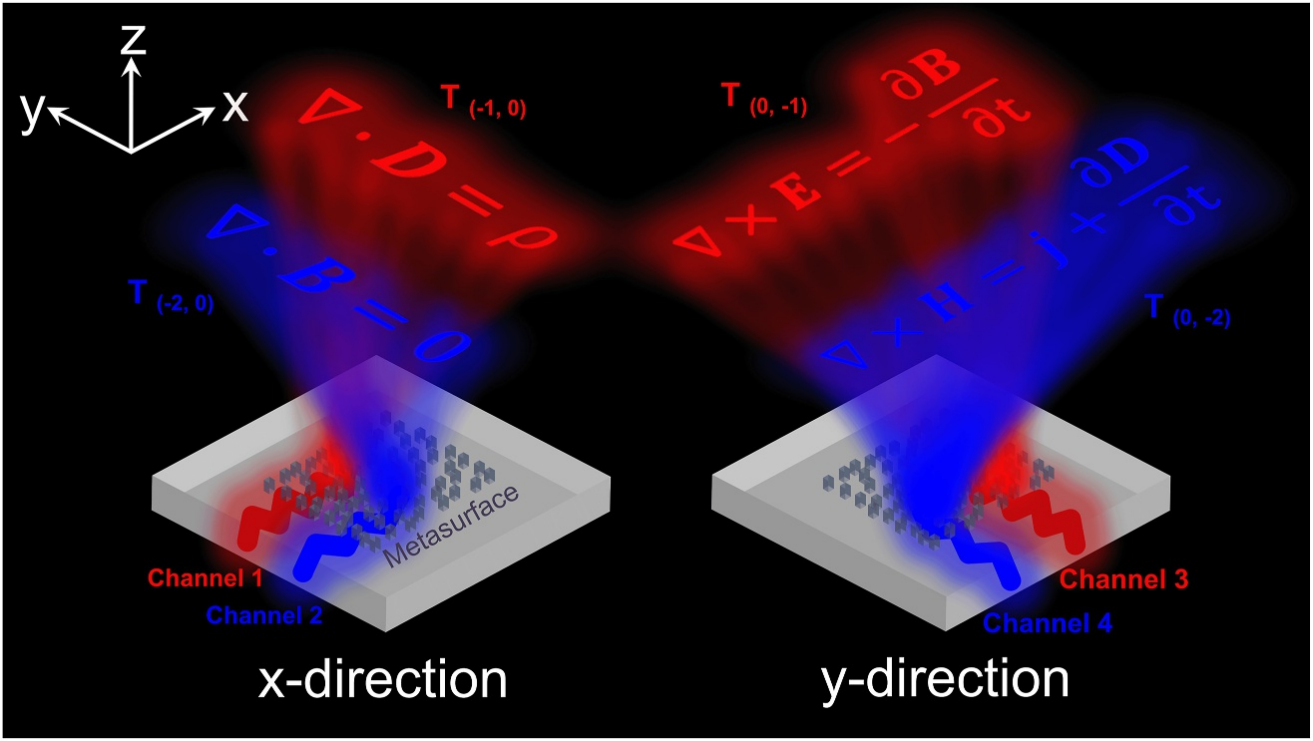
Schematic of the diffraction‐order‐multiplexed four‐channel crosstalk‐free metahologram. The device consists of a flat glass waveguide and an array of titanium dioxide (TiO_2_) nanopillars with square‐shaped cross‐sections arranged on top. Incident light propagates inside the glass waveguide via TIR and gets out‐coupled by the nanopillar array by diffraction. Four independent images (e.g., “Maxwell's four equations” in the figure) can be selectively projected into the far field depending on the diffraction order in the out‐coupling process, respectively denoted as T_(−1, 0)_, T_(−2, 0)_, T_(0, −1)_, and T_(0, −2)_.

Figure 2a illustrates the schematic of the proposed metahologram and its constituent unit cell. Without in‐plane displacements of the nanopillars along the two axes, the metahologram behaves as a two‐dimensional (2D) grating with periodicity *P* along both the *x‐* and *y*‐axes. For the multi‐channel metahologram in this study, where different images are encoded into different diffraction orders, crosstalk occurs when multiple diffraction orders are present in the out‐coupled light. To eliminate such inter‐channel crosstalk, the structural parameters of the nanopillars are optimized to ensure that only one of the four diffraction orders— T_(−1, 0)_, T_(−2, 0)_, T_(0, −1)_, and T_(0, −2)_ —is generated at a time. Additionally, to facilitate ease of device operation, the out‐coupled diffraction light is designed to emit vertically in transmission.

**FIGURE 2:**
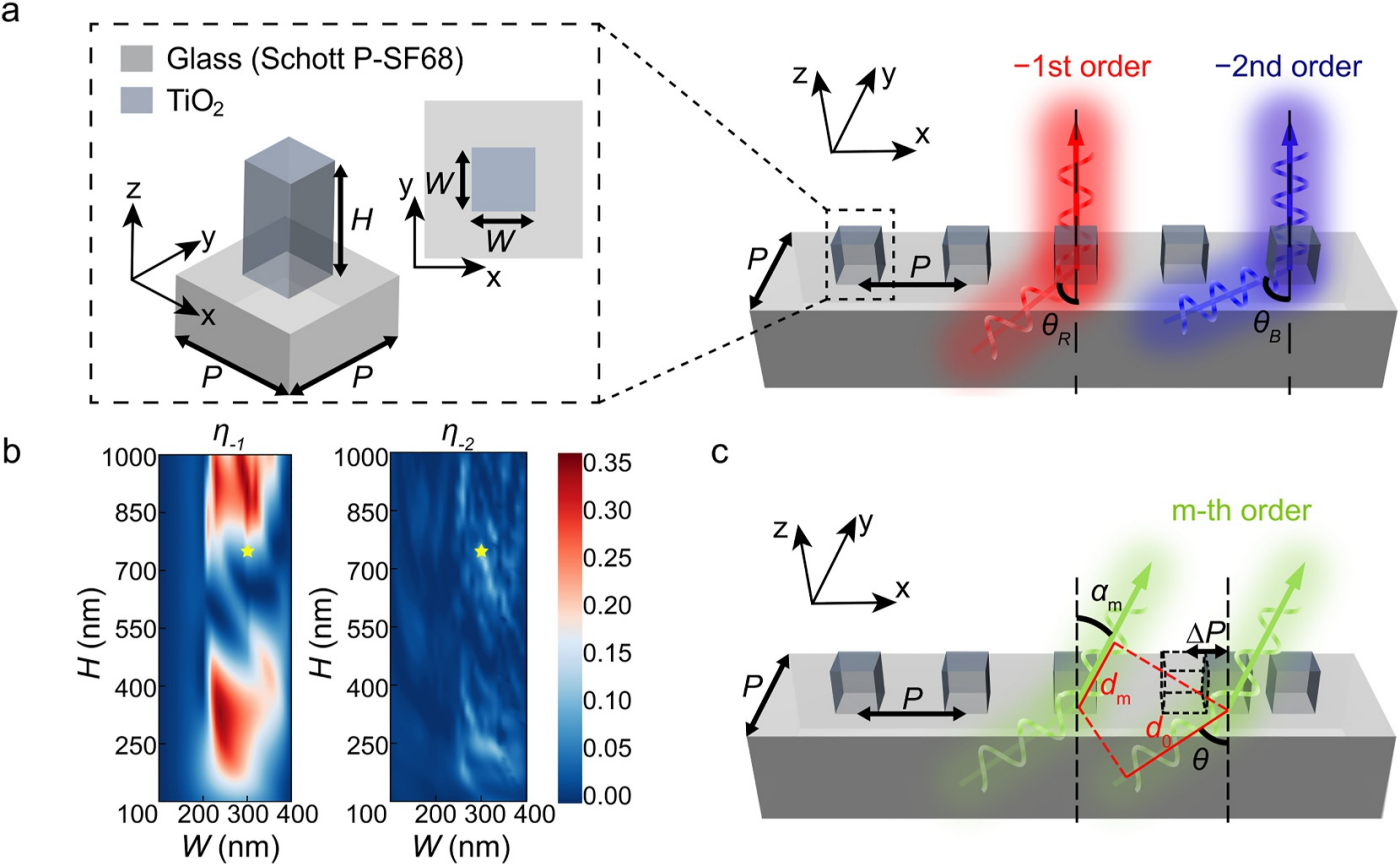
Design of the constituent nanopillar for the diffraction‐order‐multiplexed four‐channel crosstalk‐free metahologram. (a) Schematic of the metahologram, which comprises uniformly sized square‐shaped TiO_2_ nanopillars of width *W* and height *H* on a high‐refractive‐index glass (Schott P‐SF68) substrate. The metahologram has a periodicity of *P* = 405 nm along both the *x*‐ and *y*‐axes. The −1st and −2nd diffraction orders of TM‐polarized incident light, respectively, with wavelengths and TIR angles of $\lambda_{R}$ = 671 nm and $\theta_{R}$ = 56.4° and $\lambda_{B}$ = 405 nm and $\theta_{B}$ = 72.4° can be generated exclusively along the normal direction, without any crosstalk from each other. (b) Simulated diffraction efficiencies of the selected orders (*η*_−1_ and *η*_−2_) versus nanopillar's structural parameters (height *H* and width *W*). The yellow star denotes the selected structural parameters (*H* = 750 nm and *W* = 300 nm), corresponding to *η*_−1_ = 11.96% and *η*_−2_ = 12.32%, respectively. (c) Schematic of detour phase modulation by the nanopillars. Δ*P* denotes the displacement of a given nanopillar inside its lattice. θ and $\alpha_{m}$ denote the TIR angle of the incident light and the out‐coupling angle of *m*th‐order diffracted light, respectively. $d_{0}=(P+\Delta P)n_{g}\;\sin\;\theta$ and $d_{m}=(P+\Delta P)n_{0}\;\sin\;\alpha_{m}$ represent the optical path differences of the *m*th order in the glass substrate and air, respectively.

We first consider the case of one‐dimensional light diffraction. According to the grating equation (Equation (1)), the number of diffraction orders present in the out‐coupled light and the corresponding spatial angles ($\alpha_{m}$ for the *m*th order) are determined by several parameters, including the ratio between the free‐space wavelength of incident light λ and the grating period *P*, the refractive indices of the media between the grating interface (glass substrate and air in this study) *n*_*g*_ and *n*_*0*_, and the angle of incidence θ of the guided illumination light.

(1) $$\begin{array}{c} n_{0}\;\sin\;\alpha_{m}=n_{g}\;\sin\;\theta +m\frac{\lambda }{P} \end{array}$$

To operate in the visible region, we set *P* = 405 nm and choose a high‐refractive‐index glass Schott P‐SF68 (*n*_*g*_ ≈ 2.0 [43, 44]) as the glass substrate. For the −1st and −2nd transmission orders (*m* = −1 and −2), we set the corresponding free‐space wavelengths and the illumination angles as $\lambda_{R}$ = 671 nm and $\theta_{R}$ = 56.4° (red light) and $\lambda_{B}$ = 405 nm and $\theta_{B}$ = 72.4° (blue light), respectively. Both $\theta_{R}$ and $\theta_{B}$ are greater than the critical angle of TIR at the glass–air interface ($\theta_{c}$ ≈ 30°), such that the two incident beams are guided stably within the flat glass waveguide. It is worth noting that the TIR angle of the guided light within the glass waveguide slab can be controlled by properly setting the incident angle of the illuminating light at the coupling element (e.g., a prism [45, 46] or a grating coupler [47, 48]) or at the polished side facet of the glass slab [36, 49]. With these parameters, each of the two transmission orders (*m* = −1 and −2) can be exclusively generated along the normal direction to the metahologram, with no crosstalk from the other. Details of the parameter optimization process are elaborated in Supporting Information S1: Section I. Since grating diffraction along the two perpendicular axes (*x*‐ and *y*‐axes) is uncoupled, the metahologram with periodicity *P* along both the *x*‐ and *y*‐axes can provide one of the four distinct diffraction orders, T_(−1, 0)_, T_(−2, 0)_, T_(0, −1)_, and T_(0, −2)_, when the guided incident light is set to have the designed wavelength and TIR angle and illuminates the metahologram along either the *x*‐ or *y*‐direction.

To ensure high diffraction efficiencies for the four selected orders, the structural parameters of the square‐shaped nanopillars (height *H* and width *W*) are further optimized. Details of the diffraction efficiency simulation are elaborated in Supporting Information S1: Section II. Figure 2b displays the simulated diffraction efficiencies for the −1st order (*η*_−1_, left panel) and the −2nd order (*η*_−2_, right panel) for different combinations of nanopillar height *H* and width *W*. In the simulation, the guided illumination light is set to be transverse‐magnetic (TM) polarized with the corresponding wavelength and propagates along the *x*‐axis at the designed TIR angle. It is worth noting that the case of propagation along the *y*‐axis is identical as the metahologram has the same periodicity along both axes. As observed in Figure 2b, the values of *η*_−2_ are significantly lower than those of *η*_−1_ across most of structural parameter range. In light of this, we set height *H* and width *W* to 750 nm and 300 nm, respectively (marked by the yellow star in Figure 2b), such that *η*_−1_ and *η*_−2_ exhibit close values of 11.96% and 12.32%, respectively. Under this setting, the chosen value of *η*_−2_ is also close to its peak value of 13.5%.

When a given nanopillar is shifted from the lattice center with an in‐plane displacement Δ*P*, a phase retardation $\varphi_{m}$, is imparted onto the *m*th‐order diffracted light. $\varphi_{m}$ is generated due to the optical path difference of light radiating from adjacent nanopillars (Figure 2c) and can be expressed as

(2) $$\begin{array}{c} \varphi_{m}=k_{0}\left(d_{m}-d_{0}\right)=k_{0}\left(n_{0}\;\sin\;\alpha_{m}-n_{g}\;\sin\;\theta \right)\left(P+\Delta P\right), \end{array}$$

where $k_{0}$ is the free‐space wave vector, and $d_{0}=(P+\Delta P)n_{g}\;\sin\;\theta$ and $d_{m}=(P+\Delta P)n_{0}\;\sin\;\alpha_{m}$ represent the optical path differences of the *m*th order in the glass substrate and air, respectively. Based on Equations (1) and (2), the following relation can be obtained, showing that for a fixed in‐plane displacement Δ*P*, the detour phase modulation imparted onto a diffracted light is proportional to its order number *m*.

(3) $$\begin{array}{c} \varphi_{m}=m\frac{2\pi }{P}\Delta P \end{array}$$

## 3 Adaptive‐Weight Phase‐Retrieval Algorithm

We then develop an adaptive‐weight phase‐retrieval algorithm to calculate the phase profiles that are encoded into the different diffraction orders for independent holographic image projections. The algorithm is adapted from the commonly used Gerchberg‐Saxton (G‐S) algorithm and introduces adaptive weights to balance the phase profile optimizations of different orders. Compared with neural network‐based inverse design approaches, which typically require training datasets and pre‐training, the employed adaptive‐weight phase‐retrieval algorithm is based on iterative alternating projections governed by physical principles and holographic reconstruction constraints [50, 51]. This physics‐driven strategy provides high transparency and flexibility for optimizing the complex phase distributions associated with diffraction‐order multiplexing, while avoiding the need to prepare task‐specific training datasets. As depicted in Figure 3, targeting to obtain the phase profiles encoded into the two selected diffraction orders (*m* = −1 and −2), the algorithm starts by creating the light fields of the two diffraction orders on the hologram plane $U_{0}^{m}$ (*m* = −1 and −2). The phase profiles of $U_{0}^{m}$ are randomly generated and have a two‐time relation with respect to each other, and the amplitudes are set to unity. After that, $U_{0}^{m}$ of the selected orders (*m* = −1 and −2) are fed into the corresponding loops where Fourier transform and inverse Fourier transform are successively performed to update the light fields on the image plane and the hologram plane, $U_{i}^{m}$ and $U_{h}^{m}$, respectively. After each Fourier transform, the amplitudes of $U_{i}^{m}$ are replaced by the square root of the target image intensity, whereas the phases are preserved. After each inverse Fourier transform, the phases of $U_{h}^{m}$ (*m* = −1 and −2) are superposed with adaptive weights $\omega_{1}$ and $\omega_{2}$ to create a new phase $\varphi_{o}$, following $\varphi_{o}=\arg\left(\omega_{1}\;\exp\left(i\cdot \arg\left(U_{h}^{-1}\right)\right)+\omega_{2}\;\exp\left(0.5i\cdot \arg\left(U_{h}^{-2}\right)\right)\right)$. $\varphi_{o}$ (2$\varphi_{o}$) is then combined with unity value amplitude as the updated $U_{h}^{-1}$ ($U_{h}^{-2}$). The weights $\omega_{j}$ are obtained by calculating the root mean squared errors (RMSEs) between the reconstructed holographic images and the target images as follows:

(4) $$\begin{array}{c} \omega_{j}=\frac{{\mathrm{RMSE}}_{j}}{\sum_{j}{\mathrm{RMSE}}_{j}}\;\left(j=1,2\right). \end{array}$$

**FIGURE 3:**
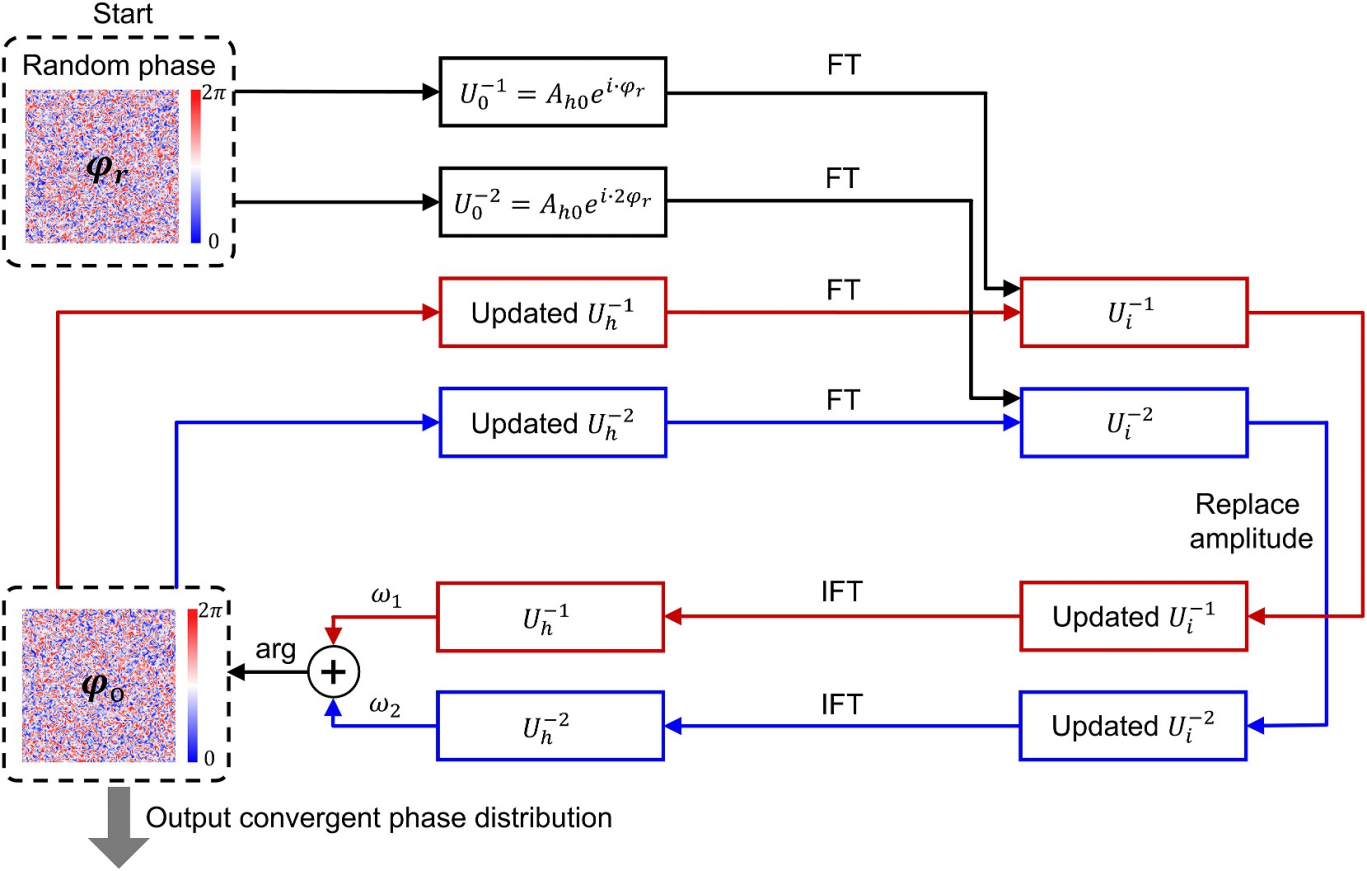
Flowchart of the proposed adaptive‐weight phase‐retrieval algorithm to calculate the phase distributions encoded into the two selected diffraction orders for independent holographic image projections. The light field on the metahologram $U_{0}^{-1}$ ($U_{0}^{-2}$) is first created by combining a random phase distribution $\varphi_{r}$ (2$\varphi_{r}$) and unity value amplitude $\left(A_{h0}=1\right)$. Then, $U_{0}^{m}$ (*m* = −1 and −2) are iteratively optimized under repeated Fourier transform (FT) and inverse Fourier transform (IFT) operations in the loops. After each Fourier transform, the amplitudes of $U_{i}^{m}$ are replaced by the square root of the target image, whereas the phases are preserved. After each inverse Fourier transform, the phases of $U_{h}^{m}$ are superposed with an adaptive weights $\omega_{1}$ and $\omega_{2}$ to create a new phase $\varphi_{o}$, which is then combined with unity value amplitude as the new $U_{h}^{m}$ (*m* = −1 and −2). The weights $\omega_{1}$ and $\omega_{2}$ are obtained by calculating the root mean squared errors (RMSEs) between the reconstructed holographic images and the target images.

Typically, the phase distributions of $U_{h}^{m}$ converge within tens of iterations, at which point the optimization loops break and produce a convergent phase distribution. To further enhance the holographic image quality, the convergent phase distribution is further optimized using a direct search algorithm [52, 53], where the element values in the phase profiles are randomly modified, and the modification will be preserved if the minimum of the two RMSE values decreases. Details of the associated phase distribution optimization process are elaborated in Supporting Information S1: Section III.

## 4 Multi‐Channel Holographic Image Projection

We first verify the developed adaptive‐weight phase‐retrieval algorithm by numerically calculating the holographic imaging results of different channels. Maxwell's four equations are employed as the target images. Using the proposed algorithm twice, we obtain the four phase profiles (all with a matrix dimension of 256 × 256), which are encoded into the four selected diffraction orders T_(−1, 0)_, T_(−2, 0)_, T_(0, −1)_, and T_(0, −2)_. The light intensity distributions on the imaging plane are then calculated using Fourier transform. As shown in Figure 4a, the four target images can be well reconstructed in the four diffraction‐order channels, all exhibiting high fidelity and being free from crosstalk. This demonstrates the high feasibility of the proposed phase‐retrieval algorithm for encoding independent images into different diffraction orders.

**FIGURE 4:**
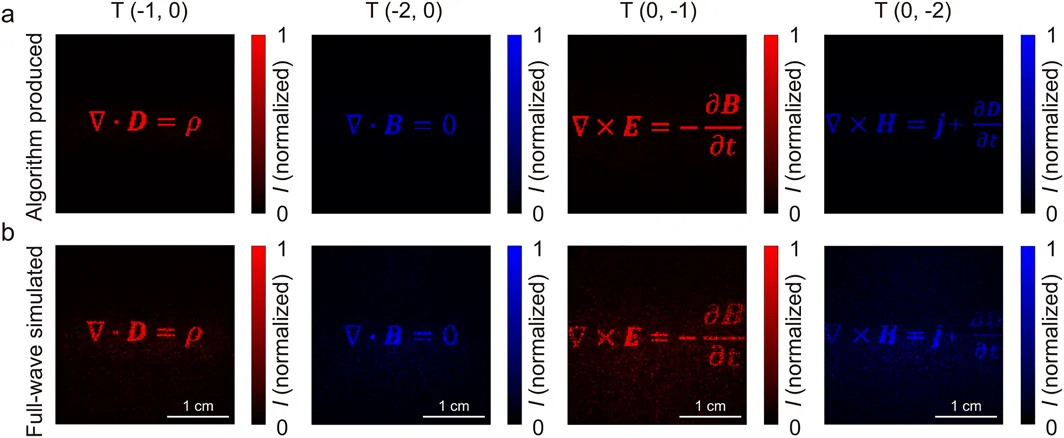
Numerical verification of the diffraction‐order‐multiplexed four‐channel crosstalk‐free metahologram. (a) Algorithm‐produced light intensity distributions of the projected holographic channels on the image plane for different diffraction orders. (b) Full‐wave simulated light intensity distributions of the projected holographic channels by the metahologram on the image plane for different diffraction orders. The image plane has a size of 3 cm × 3 cm and is located 6 cm above the metahologram plane.

We then perform a full‐wave electromagnetic simulation of the constructed diffraction‐order‐multiplexed metahologram using the finite‐difference time‐domain (FDTD) method. Consistent with the matrix size of the obtained phase modulation profiles, the metahologram consists of 256 × 256 nanopillars with the optimized structural parameters (*P* = 405 nm, *H* = 750 nm, and *W* = 300 nm). Also, based on Equation (3), each nanopillar exhibits in‐plane displacement values along both the *x*‐ and *y*‐axes in its lattice to impart the required detour phase modulation. TM‐polarized guided incident light, propagating either along the *x*‐ or *y*‐axis, is set to have the designed wavelength and TIR angle of $\lambda_{R}$ = 671 nm and $\theta_{R}$ = 56.4° for the display channels of T_(0, −1)_/T_(−1, 0)_, and $\lambda_{B}$ = 405 nm and $\theta_{B}$ = 72.4° for the display channels of T_(0, −2)_/T_(−2, 0)_, respectively. To simulate the far‐field‐projected holographic images, the light field on the metahologram plane is captured to simulate the light intensity distributions on the image plane, which is located 6 cm above the metahologram plane. Figure 4b shows the simulated imaging results of the four diffraction order channels. Four distinct, high‐fidelity, and crosstalk‐free images are formed by the out‐coupled light from each diffraction order, exhibiting good correspondence to the algorithm‐produced results. Additionally, we simulate the light intensity distribution over the full projection‐side space of the metahologram (Supporting Information S1: Section IV). The results show that all projected images lie within the designed field of view, with no additional diffraction channels that would introduce crosstalk. This further validates the intended crosstalk‐free projection functionality of the metahologram. The device operational efficiency, defined as the ratio of the projected holographic image intensity to the incident light intensity onto the metahologram, is calculated to be 11.48% for the T_(−1, 0)_ channel, 12.44% for the T_(0, −1)_ channel, 6.96% for the T_(−2, 0)_ channel, and 7.12% for the T_(0, −2)_ channel.

For the four‐channel metahologram described above, when each of the four target images is projected along the normal direction of the device plane, no other channels are generated according to the grating equation (Equation (1)), ensuring a crosstalk‐free multi‐channel holographic image projection. Due to the limited refractive index of the glass waveguide (*n*_*g*_ ≈ 2.0), the proposed waveguide‐based metahologram can support at most two normally radiating display channels (for grating diffraction along a single axis) without generating additional orders. However, when multiple orders are allowed to generate in the out‐coupled diffraction light, the proposed metahologram design can accommodate more display channels. It is worth noting that although multiple images carried by different diffraction orders are projected simultaneously in this case, they are angularly separated, making this approach suitable for cases where only the image projected along the normal direction of the device is of interest.

Based on the same strategy, we design a diffraction‐order‐multiplexed six‐channel metahologram hosting two sets of RGB display channels and demonstrate holographic projections of two independent full‐color images when the guided illumination light propagates along the *x*‐ and *y*‐axes (see Figure 5a). The red, green, and blue color components for the two target full‐color images are encoded into the −1st (T_(−1, 0)_ and T_(0, −1)_), −2nd (T_(−2, 0)_ and T_(0, −2)_), and −3rd (T_(−3, 0)_ and T_(0, −3)_) diffraction orders, respectively. To compensate for chromatic dispersion, the sizes of different color components are accordingly adjusted to be inversely proportional to the wavelengths of illumination ($\lambda_{R}$ = 671 nm, $\lambda_{G}$ = 532 nm, and $\lambda_{B}$ = 405 nm for red, green, and blue color light, respectively) [31, 32]. To achieve normal emission of the selected order, the period *P* of the meta‐atoms is set to 600 nm and the TIR angles for the guided RGB illumination light are set to $\theta_{R}$ = 34.2°, $\theta_{G}$ = 61.3°, and $\theta_{B}$ = 74.8°, respectively. The structural parameters of the TiO_2_ nanopillar (height *H* and width *W*) are optimized to *H* = 280 nm and *W* = 500 nm. The meta‐atoms provide diffraction efficiencies of *η*_−1_ = 16.59% for red light, *η*_−2_ = 8.69% for green light, and *η*_−3_ = 8.68% for blue light. Details of associated parameter optimization process are elaborated in Supporting Information S1: Section V.

Similar to the case of the four‐channel metahologram, the phase profiles encoded onto the six‐channel metahologram are first obtained using the proposed adaptive‐weight iterative algorithm, where the phase profiles imparted onto the selected three diffraction orders, following the relation governed by Equation (3), are simultaneously optimized in three parallel loops. Following this, the direct search algorithm is applied to further optimize the generated phase profiles. Details of the optimization process are provided in Supporting Information S1: Section VI.

After the optimization process, two phase profiles corresponding to the reconstructions of the two target full‐color images are obtained. The algorithm‐produced and full‐wave simulated images are generated in the same manner as in the previous case. Figure 5b,c shows the algorithm‐produced and full‐wave simulated imaging results of the two full‐color images, along with their RGB constituent components. As expected, the metahologram successfully produces two distinct full‐color images, formed by overlapping different color components under the illumination of guided incident light with the designed wavelengths and TIR angles, propagating along either the *x*‐ or *y*‐axis. The recorded images exhibit remarkable fidelity to the original encoded ones and align closely with the design intent. Additionally, we simulate the light intensity distribution over the full projection‐side space of the metahologram (Supporting Information S1: Section VII). The results show that all projected images lie within the designed field of view, whereas any additional diffraction channels appear only at larger angular locations and therefore introduce no crosstalk into the formed holographic images. This further confirms the intended design of the device. The device operational efficiency, defined as the ratio of the projected holographic image intensity to the incident light intensity onto the metahologram, is calculated to be 9.48% for the T_(−1, 0)_ channel, 6.87% for the T_(0, −1)_ channel, 4.78% for the T_(−2, 0)_ channel, 3.90% for the T_(0, −2)_ channel, 3.98% for the T_(−3, 0)_ channel, and 3.52% for the T_(0, −3)_ channel.

**FIGURE 5:**
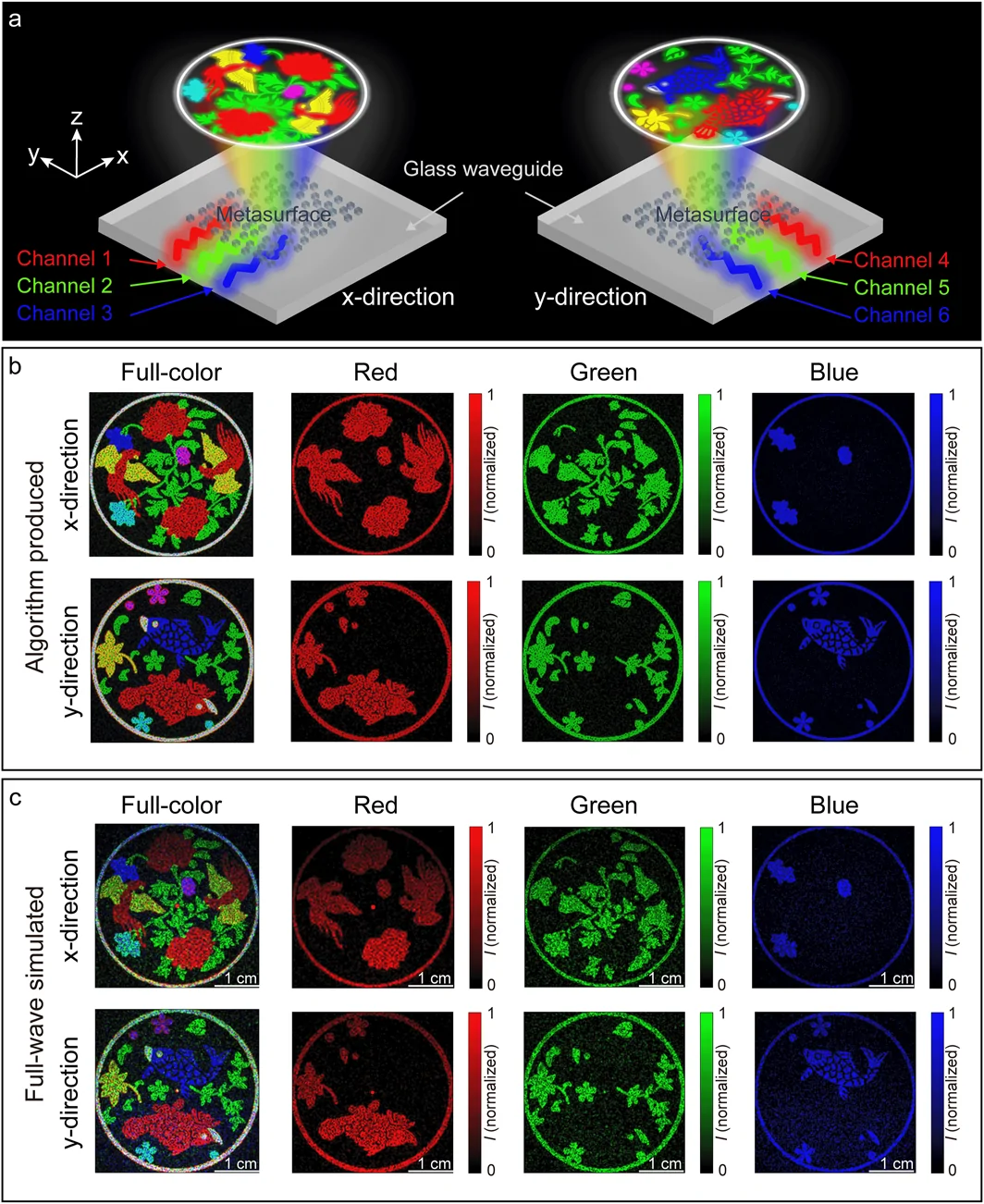
Waveguide‐based six‐channel metahologram for two full‐color image projection. (a) Schematic of the waveguide‐based six‐channel metahologram for two full‐color image projection. Depending on the propagation direction of the guided illumination light (along either the *x*‐ or *y*‐axis), the metahologram projects one of the two distinct full‐color images formed by overlapping RGB color components in air. (b–c) Algorithm‐produced (b) and full‐wave simulated (c) full‐color holographic images as well as their RGB color components. The image plane has a size of 4.05 cm × 4.05 cm and is located 12 cm above the metahologram plane.

## 5 Conclusion

In conclusion, we present a new class of waveguide‐based multi‐channel metaholograms that leverage diffraction‐order multiplexing to achieve crosstalk‐free holographic image projection. By controlling the wavelength, angle, and propagation direction of the guided illumination light, the metahologram selectively projects four independent images without inter‐channel interference. To realize the diffraction‐order‐multiplexed phase encoding, we develop an adaptive‐weight phase‐retrieval algorithm that simultaneously optimizes the phase profiles for different diffraction orders. Using the same design strategy, we further demonstrate a six‐channel waveguide metahologram capable of projecting two full‐color images. In the present design, the metahologram employs isotropic square‐shaped meta‐atoms. Beyond this configuration, anisotropic meta‐atoms could be introduced so that spin‐dependent manipulation or the Pancharatnam‐Berry (geometric) phase can be potentially combined with the detour phase to provide additional degrees of freedom and further increase the number of independent channels [54, 55, 56]. Compared with existing multichannel approaches, our waveguide‐based design offers more display channels, eliminates inter‐channel crosstalk, and suppresses directly transmitted residual light by confining it within the waveguide. This work provides a novel pathway for metahologram development with enhanced information capacity and improved image fidelity, opening new opportunities for compact display and imaging applications.

## Author Contributions

**Zhiyu Ning:** conceptualization, investigation, validation, formal analysis, data curation, visualization, writing – original draft. **Zeyang Liu:** conceptualization, methodology, investigation, formal analysis, data curation, funding acquisition, writing – original draft, project administration, supervision. **Deqian Zheng:** investigation, data curation, formal analysis. **Niu Liu:** investigation, data curation. **Hao Gao:** investigation. **Tianzhang Peng:** investigation, data curation. **Ziqian Xi:** visualization. **Xinliang Zhang:** formal analysis, supervision. **Cheng Zhang:** conceptualization, project administration, funding acquisition, writing – review and editing, supervision, resources.

## Conflicts of Interest

The authors declare no conflicts of interest.

## Supporting information


Supporting Information S1

## Data Availability

The data that support the findings of this study are available from the corresponding author upon reasonable request.
