# Supplementary material for: Waveguide‐Based Four‐Channel Crosstalk‐Free Metahologram Multiplexed by Diffraction Orders
Source: Nanophotonics. 2026 Apr 25;15(9):e70078. doi: 10.1002/nap2.70078 (PMC13642528; doi:10.1002/nap2.70078)
Supplement: Supplementary file 1 [file NAP2-15-e70078-s001.pdf]

Supplementary Information for

Waveguide-based four-channel crosstalk-free metahologram multiplexed by diffraction orders

Zhiyu Ning,[1,†] Zeyang Liu,[1,†,*] Deqian Zheng,[1] Niu Liu,[2] Hao Gao,[1] Tianzhang Peng,[1] Ziqian Xi,[1] Xinliang Zhang,[2] and Cheng Zhang[1,2,*]

[1]*School of Optical and Electronic Information, Huazhong University of Science and Technology, Wuhan 430074, China*

[2]*Wuhan National Laboratory for Optoelectronics, Huazhong University of Science and Technology, Wuhan 430074, China*

[†]*These authors contributed equally.*

* *zeyangliu@hust.edu.cn*, *cheng.zhang@hust.edu.cn*

This Supplementary Information includes:

Section I. Device parameter optimization of the four-channel crosstalk-free metahologram

Section II. Diffraction efficiency simulation of the meta-atom array

Section III. Direct search algorithm for phase profile optimization

Section IV. Light intensity distribution over the full projection-side space of the four-channel metahologram

Section V. Device parameter optimization of the six-channel metahologram for two full-color image projections

Section VI. Phase profile generation for the six-channel metahologram

Section VII. Light intensity distribution over the full projection-side space of the six-channel metahologram

Section I: Device parameter optimization of the four-channel crosstalk-free metahologram

To eliminate the crosstalk between different diffraction-order channels, we optimize the structural parameters in the device design to ensure that only one of the four diffraction orders, $T_{(-2,0)}$, $T_{(-1,0)}$, $T_{(0,-1)}$, and $T_{(0,-2)}$, is generated at a time. Consider a one-dimensional (1D) grating with periodicity P along the *x*-direction. To ensure that only the target diffraction orders (m = -1, -2) are out-coupled normal to the device plane in transmission, while other orders are suppressed, the following equations, derived from Eq. (1), must be satisfied.

$$\begin{cases} n_g sin\theta_R - \frac{\lambda_R}{P} = 0 \\ n_g sin\theta_R - 2\frac{\lambda_R}{P} \leq -n_0 \end{cases} \qquad (S1)$$

$$\begin{cases} n_g sin\theta_B - 2\frac{\lambda_B}{P} = 0 \\ n_g sin\theta_B - \frac{\lambda_B}{P} \geq n_0 \\ n_g sin\theta_B - 3\frac{\lambda_B}{P} \leq -n_0 \end{cases} \qquad (S2)$$

After simplification of the equations, we have

$$\begin{cases} n_g sin\theta_R = \frac{\lambda_R}{P} \geq n_0 \\ n_g sin\theta_B = 2\frac{\lambda_B}{P} \geq 2n_0 \end{cases}. \qquad (S3)$$

To operate in the visible region, we set λ_R = 671 nm and λ_B = 405 nm. Eq. (S3) indicates that the n_g at λ_B should be greater than 2, thus we select Schott P-SF68 glass as the substrate, which has a refractive index of approximately 2.10 at 405 nm (see Figure S1). With the above conditions, the grating period P is set to 405 nm, and we can obtain θ_R = 56.4° and θ_B = 72.4° accordingly. As grating diffraction along the two perpendicular axes (*x*- and *y*-axes) is uncoupled, the metahologram with periodicity P along both the *x*- and *y*-axes can provide one of the four distinct diffraction orders, $T_{(-2,0)}$, $T_{(-1,0)}$, $T_{(0,-1)}$, and $T_{(0,-2)}$, when the guided incident light is set to have the designed wavelength and TIR angle and illuminates the metahologram along either the *x*- or *y*-direction.

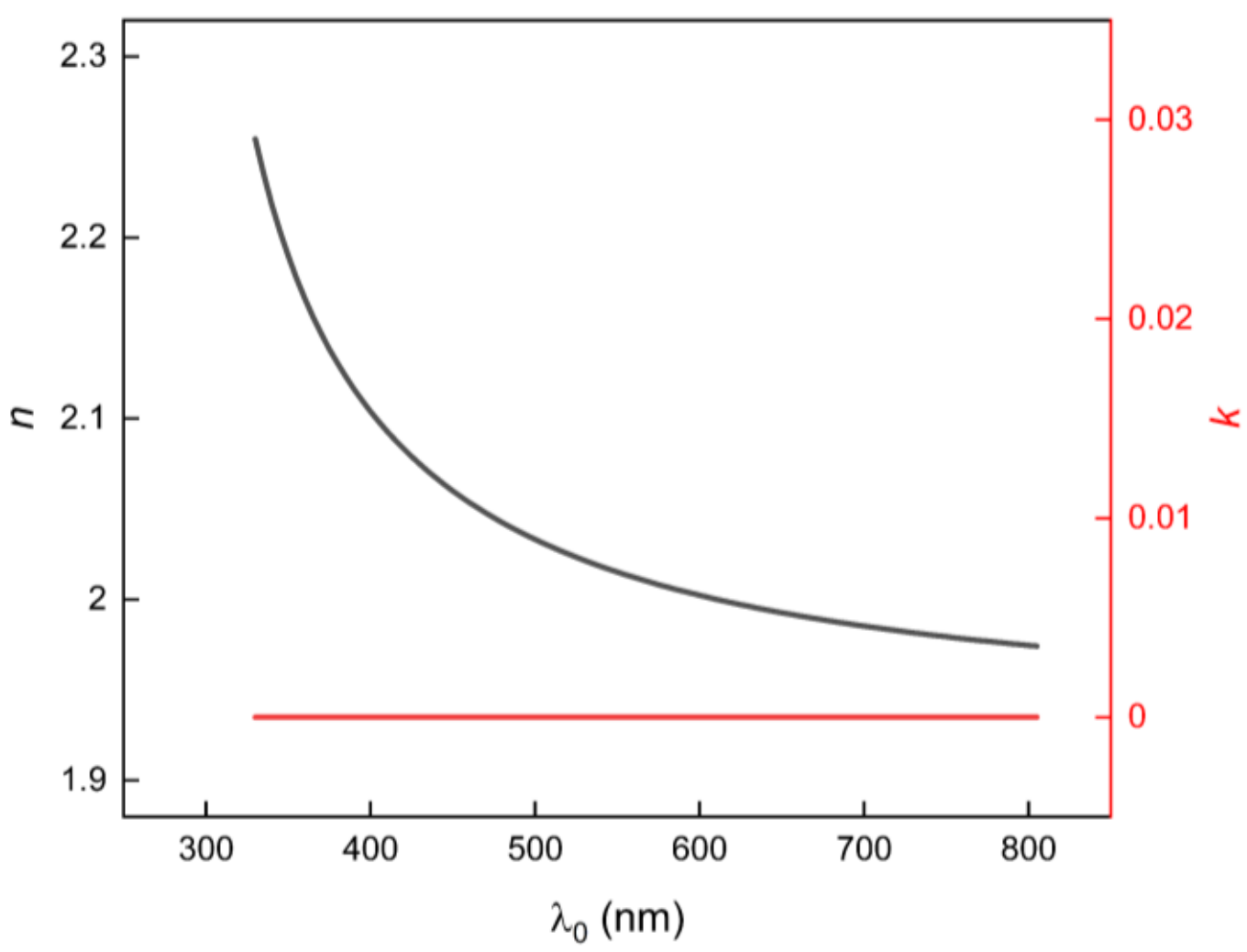

Figure S1. Refractive index *n* and extinction coefficient *k* of Schott P-SF68 glass across the visible range [1], [2].

Section II: Diffraction efficiency simulation of the meta-atom array

Diffraction efficiency of meta-atom array is simulated using the open-source solver RETICOLO, which is based on Rigorous Coupled-Wave Analysis (RCWA) algorithm [3],[4]. Compared with the finite-difference time-domain (FDTD) method, RETICOLO provides significantly higher computational efficiency, thereby facilitating extensive parameter sweeps during the metasurface design process. All computations are performed with Fourier components ranging from -10 to 10. In diffraction efficiency simulation, the meta-atoms have a fixed pitch P = 405 nm. The width W and height H of the TiO_2 nanopillar vary within specific ranges from 50 nm to 400 nm and 100 nm to 1000 nm, respectively, with an interval of 10 nm. The incident light is set to propagate along the x-axis inside the glass slab waveguide and illuminate the meta-atom with the designed wavelength and incident angle. With different structural parameters of the nanopillar and polarization states of guided incident light (TE and TM), diffraction efficiencies η_{-1} and η_{-2} of the selected orders m = -1 & -2 are obtained.

To realize balanced efficiencies for both diffraction orders, a figure of merit (FoM) is defined as:

$$FoM = \min\{\eta_{-1}, \eta_{-2}\}. \qquad (S4)$$

The maximum value of FoM for TM and TE polarization reaches 11.96% and 8.87%, respectively, which is denoted by a yellow star (H = 750 nm, W = 300 nm) and a red circle (H = 960 nm, W = 300 nm) in Figure S2. Therefore, we set the polarization of guided incident light to be TM polarization and set height H and width W to 750 nm and 300 nm in device design to ensure reasonable working efficiency.

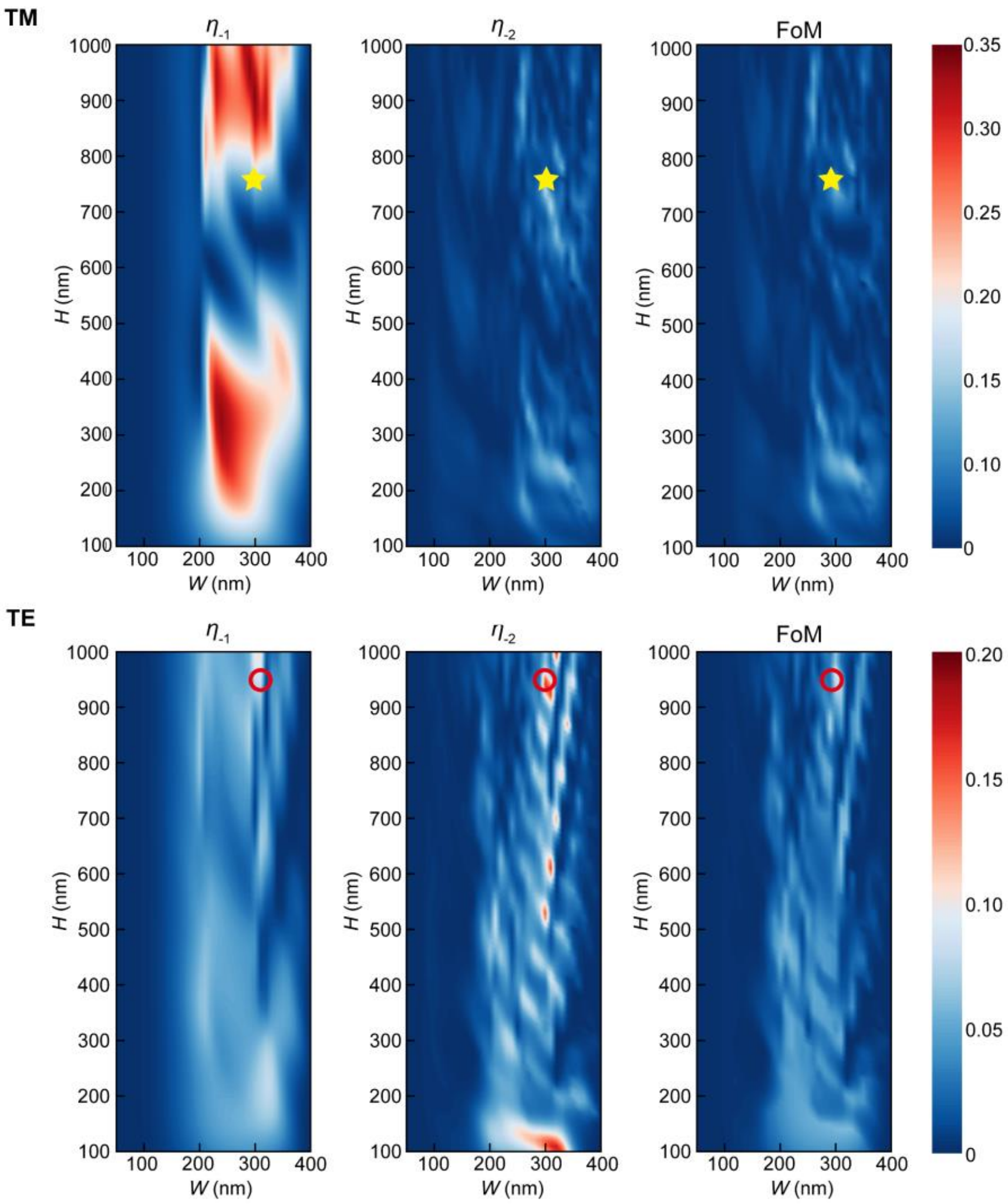

Figure S2. Simulated diffraction efficiencies (η_{-1} and η_{-2}) and FoMs for different structural parameters (W and H) of the meta-atom and different polarizations (TM and TE) of the guided incident light. Yellow star and red circle mark the maximum values of FoM for TM and TE polarizations, which reach 11.96% and 8.87%, respectively. The structural parameters corresponding to the maximum values of FoM are H = 750 nm, W = 300 nm, and H = 960 nm, W = 300 nm for TM and TE polarizations, respectively.

Section III: Direct search algorithm for phase profile optimization

To improve the holographic image quality, we use a direct search algorithm to further optimize the phase profile generated by the proposed adaptive-weight iterative algorithm (see Figure S3). The direct search algorithm optimizes the convergent phase profile iteratively. In each iteration, a single element value within the phase profile is randomly selected and modified. The modification is retained if it results in an improvement in the holographic image quality. We define a loss function $LOSS$ as a metric for evaluating imaging degradation for the multi-channel holographic imaging.

$$LOSS = \max\{RMSE_j\}\ (j = 1, 2, \dots, N) \qquad (S5)$$

$RMSE_j$ denotes the root mean squared error between the reconstructed holographic image and the target image in each channel j, and N is the total number of channels. By minimizing the maximum value of $RMSE_j$, the worst-performing channel can be improved preferentially, such that we can achieve high image quality for all different channels.

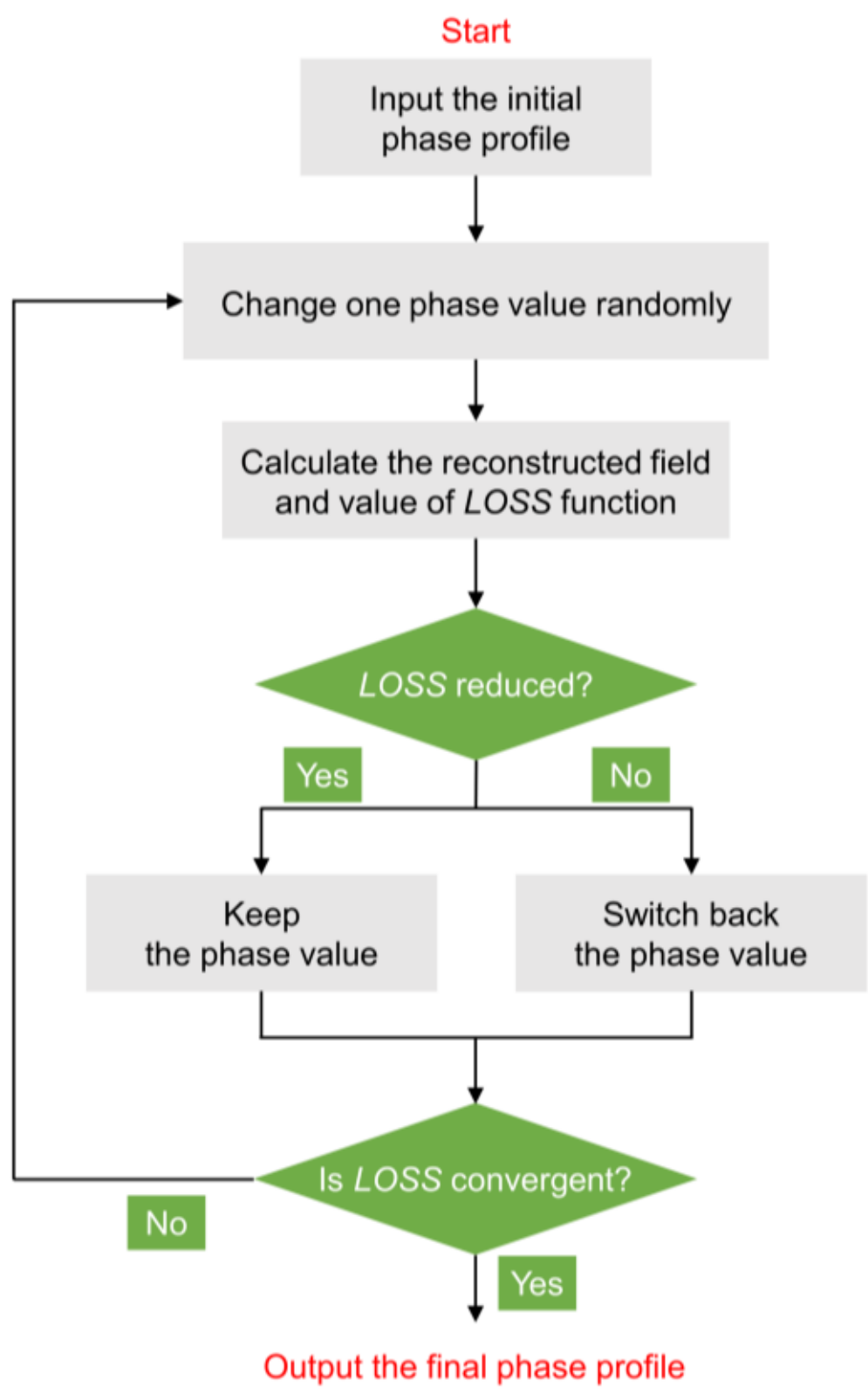

Figure S3. Flowchart of the direct search algorithm adopted to optimize the phase profile.

Section IV: Light intensity distribution over the full projection-side space of the four-channel metahologram

We simulate the light intensity distribution over the full projection-side space of the four-channel metahologram (Fig. S4). The results show that all target images are projected along the surface normal within the designed 14° field of view and that no additional diffraction channels are present that would introduce crosstalk into the formed images. This further validates the intended crosstalk-free projection functionality of the metahologram.

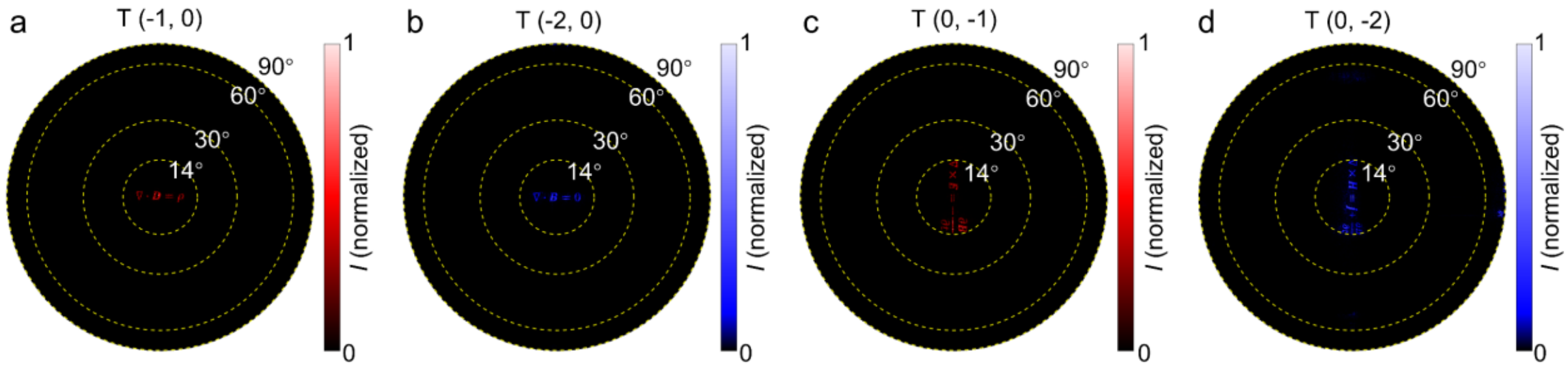

Figure S4. Normalized far-field projection patterns of the four-channel metahologram. The yellow dashed circles of different radii indicate polar angles of 14°, 30°, 60°, and 90°.

Section V: Device parameter optimization of the six-channel metahologram for two full-color image projections

For the proposed waveguide-based two-channel full-color metahologram, three diffraction orders (m = -1, -2, -3) are employed, corresponding to the projection of red, green, and blue image component, respectively. To generate these three orders exclusively, Eq. (S6) should be satisfied, which requires that the refractive index of the glass waveguide has to be greater than 3. This requirement cannot be achieved for current glass materials, and thus these three diffraction orders cannot be exclusively generated and emitted along the normal direction of the device.

$$\begin{cases} n_g sin\theta_R = \dfrac{\lambda_R}{P} \geq n_0 \\ n_g sin\theta_G = \dfrac{2\lambda_G}{P} \geq 2n_0 \\ n_g sin\theta_B = \dfrac{3\lambda_B}{P} \geq 3n_0 \end{cases} \qquad (S6)$$

We now allow the generation of multiple orders in diffraction and only consider the condition that the three diffraction orders are normally emitted from the waveguide, which can be expressed as:

$$\begin{cases} n_g sin\theta_R = \dfrac{\lambda_R}{P} \geq n_0 \\ n_g sin\theta_G = \dfrac{2\lambda_G}{P} \geq n_0 \, . \\ n_g sin\theta_B = \dfrac{3\lambda_B}{P} \geq n_0 \end{cases} \qquad (S7)$$

To satisfy the conditions, we set the wavelengths of red, green, and blue light to λ_R = 671 nm, λ_G = 532 nm, and λ_B = 405 nm, and set the period as P = 600 nm. Using the same glass SCHOTT P-SF68 as the waveguide material, we can obtain the corresponding incident angles as: θ_R = 34.20°, θ_G = 61.34°, and θ_B = 74.81°. The diffraction efficiencies of the meta-atom with different structural parameters (width W and height H) under TM and TE polarized light illumination are simulated (see Figure S6). In simulation, width W and height H of the meta-atom vary from 50 nm to 550 nm and 100 nm to 1000 nm, respectively, with an interval of 10 nm. The FoM is defined as:

$$FoM = \min\{\eta_{-1}, \eta_{-2}, \eta_{-3}\}. \quad (S8)$$

The maximum value of FoM for TM and TE polarization reaches 8.68% and 7.54%, respectively, which is denoted by a yellow star (H = 280 nm, W = 500 nm) and a red circle (H = 180 nm, W = 400 nm) in Figure S5. Therefore, we set the polarization of guided incident light to be TM polarization and set height H and width W to 280 nm and 500 nm in device design to ensure reasonable working efficiency.

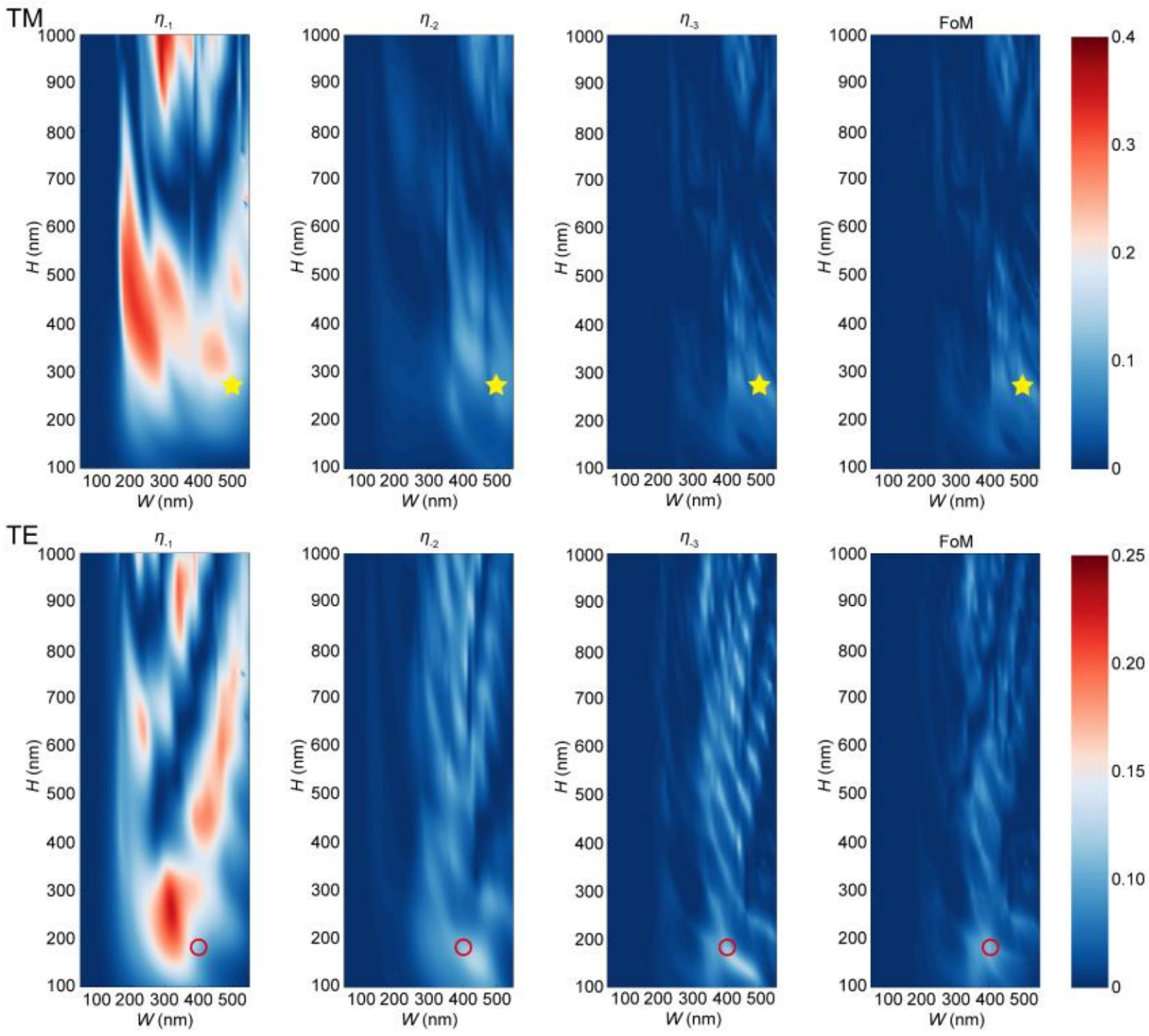

Figure S5. Simulated diffraction efficiencies (η_{-1}, η_{-2}, and η_{-3}) and FoMs for different structural parameters (W and H) of the meta-atom and different polarizations (TM and TE) of the guided incident light. Yellow star and red circle mark the maximum values of FoM for TM and TE polarizations, which reach 8.68% and 7.54%, respectively. The structural parameters corresponding to the maximum values of FoM are H = 280 nm, W = 500 nm, and H = 180 nm, W = 400 nm for TM and TE polarizations, respectively.

Section VI: Phase profile generation for the six-channel metahologram

The proposed adaptive-weight iterative algorithm is modified to calculate the phase profile for the two-channel full-color metahologram, where three diffraction orders (*m* = -1, -2, -3) are employed. Flow chart of the algorithm is depicted in Figure S6. The phase profiles imparted onto the three selected diffraction orders are randomly generated following the relation governed by Eq. (3). They are optimized in three parallel loops simultaneously, each with an adaptive weight (ω_1, ω_2, and ω_3). Fourier transform (FT) and inverse Fourier transform (IFT) are successively performed to update the light fields on the image plane and hologram plane, U_i^m and U_h^m, respectively.

The update rule for the phase profile in each iteration is given by

$$\varphi_h = arg\left(\omega_1 \exp\left(i \cdot arg(U_h^{-1})\right) + \omega_2 \exp\left(i \cdot \frac{arg(U_h^{-2})}{2}\right) + \omega_3 \exp\left(i \cdot \frac{arg(U_h^{-3})}{3}\right)\right). (S9)$$

The generated phase profile is also further optimized by using the direct search algorithm described in Section III, Supplementary Information. Implementing this algorithm twice, we obtain the two phase profiles corresponding to the reconstructions of two target full-color images.

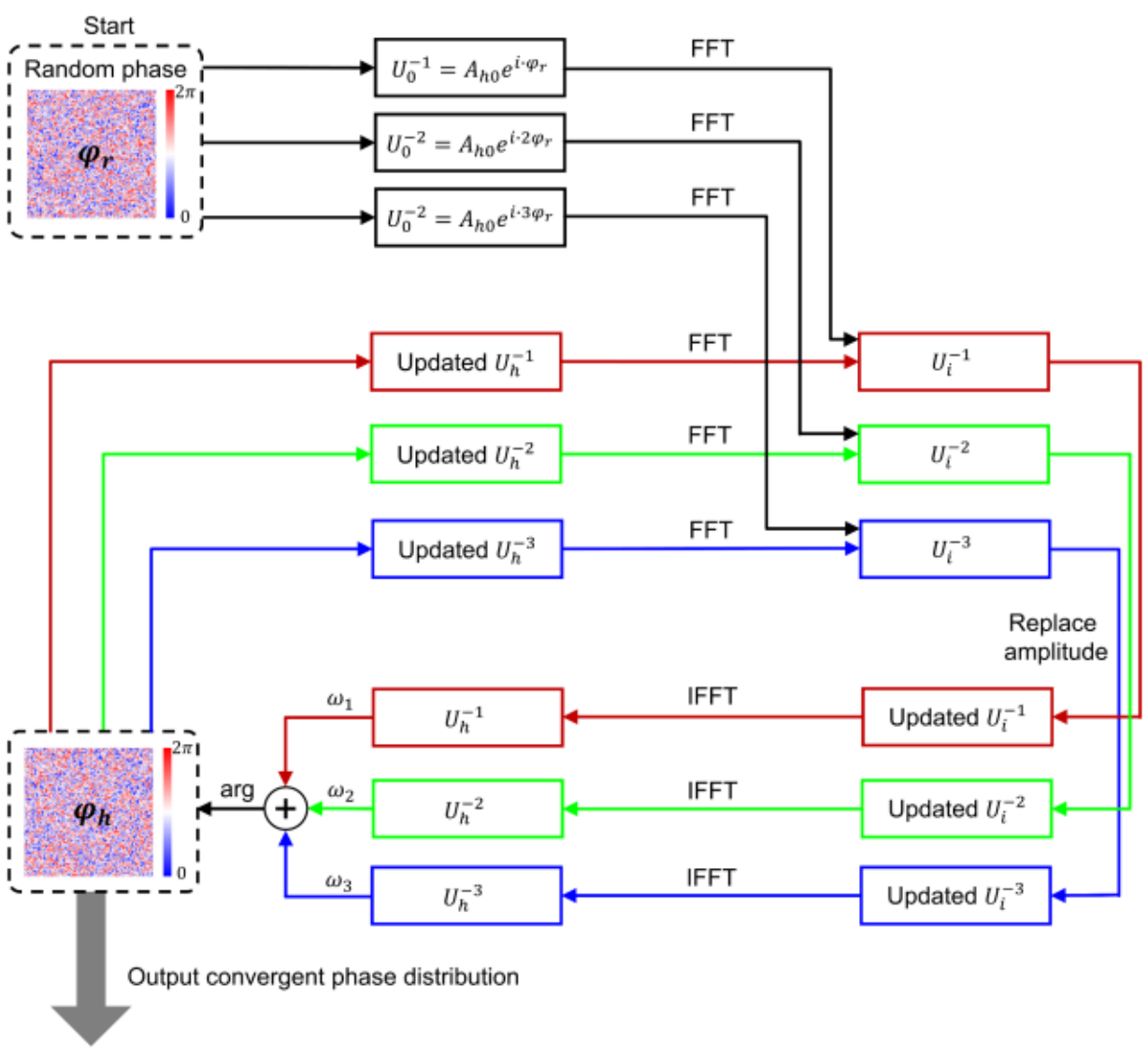

Figure S6. Flow chart of the proposed adaptive-weight phase-retrieval algorithm to calculate the phase distributions encoded into the three selected diffraction orders for independent holographic image projections.

Section VII: Light intensity distribution over the full projection-side space of the six-channel metahologram

We simulate the light intensity distribution over the full projection-side space of the six-channel metahologram (Fig. S7). The results show that all target images are projected along the surface normal within the designed 10° field of view, while any additional diffraction channels appear only at larger polar angles and therefore introduce no crosstalk into the formed holographic images. This angular separation ensures that the quality of the central target images remains unaffected, maintaining a crosstalk-free display within the designed field of view.

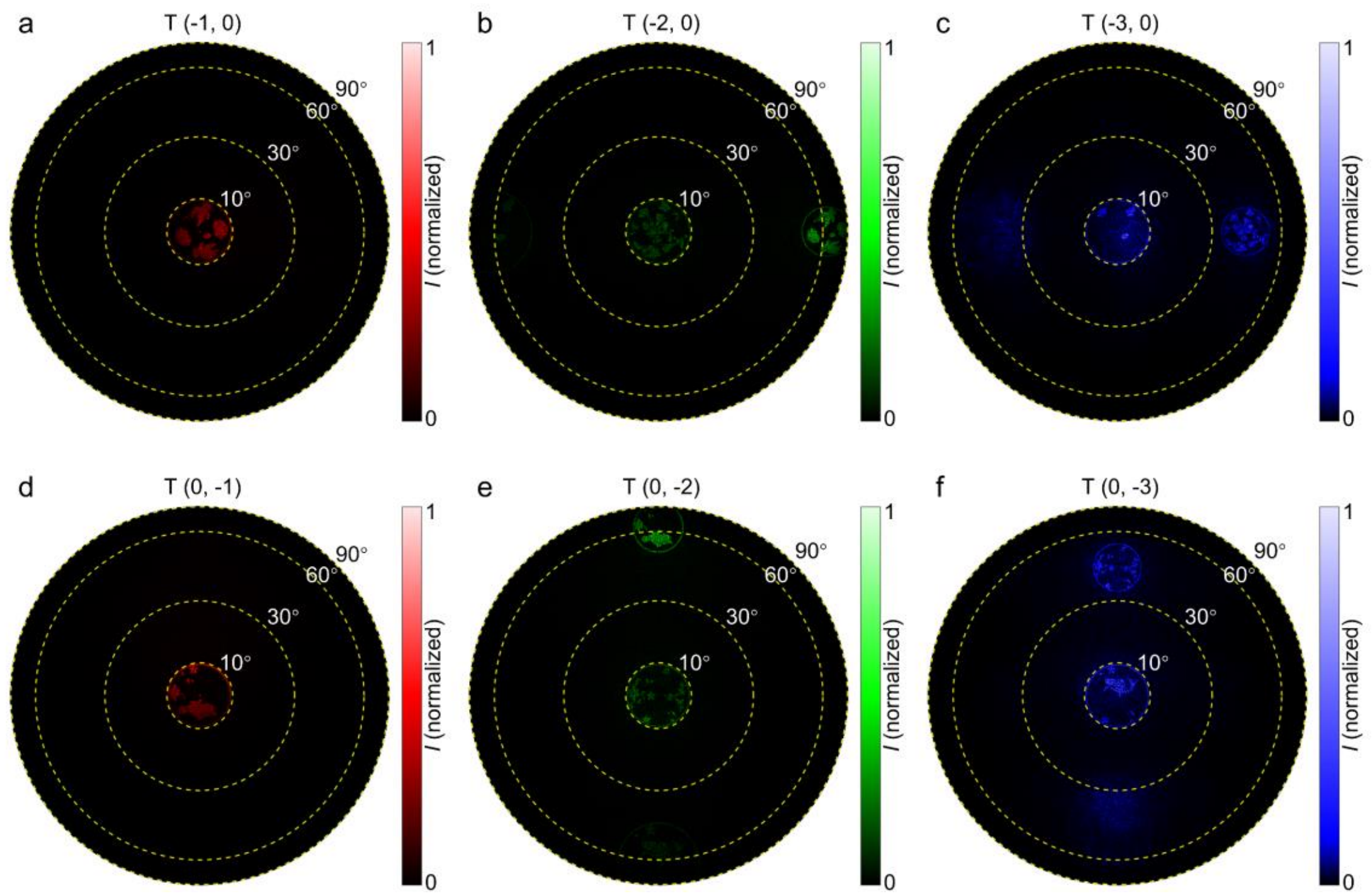

Figure S7. Normalized far-field projection patterns of the six-channel metahologram. The yellow dashed circles of different radii indicate polar angles of 10°, 30°, 60°, and 90°.

References

[1] https://www.schott.com/shop/advanced-optics/zh/Optical-Glass/P-SF68/c/glass-P-SF68

[2] https://www.filmetrics.com/refractive-index-database/Schott+P-SF68

[3] Moharam, E. B. Grann, D. A. Pommet, and T. K. Gaylord, "Formulation for stable and efficient implementation of the rigorous coupled-wave analysis of binary gratings," *Journal of the Optical Society of America A* 12, no. 5 (1995): 1068-1076. https://doi.org/10.1364/JOSAA.12.001068

[4] Hugonin and P. Lalanne, "RETICOLO software for grating analysis," (2021). https://doi.org/10.48550/arXiv.2101.00901
